# Genotoxic Biomonitoring in Children Living near the El Fraile Mine Tailings in Northern Guerrero State, Mexico

**DOI:** 10.3390/toxics10110674

**Published:** 2022-11-08

**Authors:** María Elena Calderon-Segura, Alejandro Ramírez-Guzmán, Oscar Talavera-Mendoza, Yolanda Carbajal-López, María del Carmen Martínez-Valenzuela, Martha Elena Mora-Herrera, Liliana Salinas-Alcántara, Patricia Hurtado-Brito

**Affiliations:** 1 Laboratorio de Toxicología Ambiental, Departamento de Ciencias Ambientales, Instituto de Ciencias de la Atmósfera y Cambio Climático, Universidad Nacional Autónoma de México, Ciudad Universitaria Coyoacán, Ciudad de México 04510, Mexico; 2 Escuela Superior de Ciencias de la Tierra, Universidad Autónoma de Guerrero, Ex Hacienda de San Juan Bautista s/n, Taxco el Viejo 40323, Mexico; 3 Facultad de Ciencias Químico Biológicas, Universidad Autónoma de Guerrero, Av. Lázaro Cárdenas s/n, Chilpancingo de los Bravo 39087, Mexico; 4 Instituto de Investigaciones en Ambiente y Salud, Universidad Autónoma de Occidente, Boulevar Macario Gaxiola, Carretera Internacional, Los Mochis 81200, Mexico; 5 Laboratorio de Fisiología y Biotecnología Vegetal, Centro Universitario Tenancingo, Universidad Autónoma, Tenancingo 52400, Mexico

**Keywords:** DNA damage, exfoliated buccal mucosa cells, urinary 8-OHdG adduct, children, El Fraile Taxco mine tailings, México

## Abstract

A genotoxic study was conducted with 101 elementary school children (56 girls and 45 boys) in the 6–7, 8–9, and 10–12 age ranges from El Fraile rural community, which is located beside the El Fraile mine tailings in Taxco of Alarcon City, in northern Guerrero State, Mexico. For this, we used the alkaline comet assay in exfoliated buccal mucosa cells, scoring three genotoxic parameters: tail intensity, tail moment, and tail length. Additionally, we detected oxidative DNA damage through urinary 8-OHdG levels by enzyme-linked immunosorbent assay. We also evaluated a control group consisting of 101 children in the same age ranges from Chilpancingo City, Guerrero, who had never lived near mining zones. Genotoxic results showed that there was a significant increase in three genotoxic parameters and urinary 8-OHdG levels in the exposed children group compared with the control group. Analysis of MANOVA revealed that boys aged 8 and 9 years had higher DNA damage than girls from the same exposure group, and Spearman’s analysis identified a positive correlation between DNA damage and sex and age. This study provides the first valuable genotoxic data in children living in areas with environmental pollution.

## 1. Introduction

**Figure 1:**
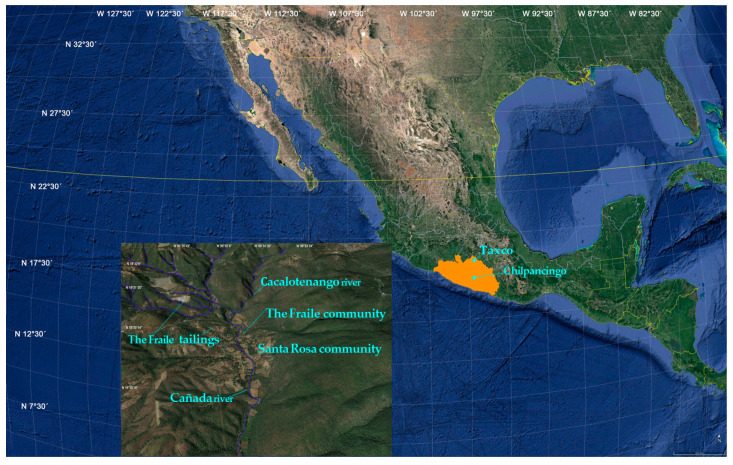
Study areas. The El Fraile community is located beside the El Fraile mine tailings in Taxco of Alarcon City in Northern, and Chilpancingo City in South, Guerrero State, México.

The Taxco Mining District in southern Mexico was one of the richest producers of precious metals (Ag and Au) in the Americas during colonial (1523–1810) times. Throughout much of the 20th Century, Taxco mines were renowned for their production of base metals (Cu, Pb, and Zn) and Ag. More than 450 years of intensive mining activity has resulted in the discharge of large quantities of tailings and mining wastes into the surrounding areas, and some of these tailings are located within communities (Santa Rosa, Dolores, and El Fraile) (Figure 1) [1,2]. The El Fraile rural community, which is located beside the El Fraile mine tailings and near the abandoned El Fraile mine, is 15 km from Taxco of Alarcon City, in northern Guerrero State, Mexico (Figure 1). In this community, people occasionally use leachates as an alternative source of domestic water during the dry (winter/spring) seasons. Furthermore, edible plants such as tomatoes, peppers, and corn for human consumption are grown in the local soil, and animals also consume plants that grow near El Fraile tailings, increasing the health risk in the trophic chain [1,2] (Figure 1). Many studies in this area have indicated that the water, soil, and plants contain heavy metals such as Ni, Hg, Pb, As, and Cd, and other elements such as Fe, Mn, and Cu [1,2]. High heavy metal levels in human urine [3,4], vegetal tissues, soil [1], and water [5] clearly indicate high exposure of the general population. However, developing children are the most sensitive population to the adverse effects of these heavy metals [6]. Children living near or on mining residue are routinely exposed to a large number of genotoxic heavy metals by inhalation, dermal absorption, drinking water, and contaminated food [7].

Different scientific studies have suggested that certain modifications in the central nervous system may also originate during early development by exposure to heavy metals [8,9,10]. Neural disorders in adults have been linked to environmental factors acting in the preconceptual, embryonic, fetal, and infantile phases of life [9,10]. The impact of such influences early in life may be partially mediated through oxidative stress that involves DNA damage and other biochemistry and molecular mechanisms [11,12]. Other studies point to a relationship between exposure to heavy metals and an increased risk of cancer [13] in the lungs [14] and in the liver [15], among others [11].

Heavy metals directly or indirectly exert their toxicity by producing a large quantity of reactive nitrogen species (RNS) or reactive oxygen species (ROS), such as hydroxyl and superoxide radicals, as well as nonradical species such as H_2_O_2_. [16,17]. Among these, hydroxyl radicals are the most reactive species; thus, they can chemically attack DNA, causing strand breaks, and oxidation of their bases gives rise to a range of oxidative DNA damage products, including 8-hydroxyadenine, 5,6-dihydroxyuracil, 5-hydroxycytosine, and several others [18].

One lesion of this type is 8-hydroxy-deoxyguanosine (8-OHdG), which is formed by the reaction of the hydroxyl radical at the C-8 position of the guanine on DNA; 8-OHdG is excised by constitutive enzymatic repair systems, appears in the blood, and is subsequently excreted in the urine [19]. The 8-OHdG level in the blood or urine is measured as a marker of oxidative DNA damage induced by environmental pollution [20,21]. It is associated with the development of various diseases, tumors, and cancers. Urinary 8-OHdG, in particular, has been the measurement most frequently used to indicate the extent of oxidative DNA damage and is an excellent molecular biomarker for mutagenic and/or carcinogenic agents. Furthermore, 8-OHdG can pair with both adenine and cytosine, but if an adenine and guanine (A:G) mismatch is not repaired, there will be a transversion of regular pairs adenine and timidine (A:T), and cytosine and guanine (C:G) [22,23].

Monitoring of biological effects as a measure of the internally effective dose is relevant for the assessment of ultimate health risks, such as cancer [24]. Many biomarkers, such as micronuclei [4], sister chromatid exchanges [25], and alkaline comet assays, have been developed to estimate exposure and to assess the risk of adverse health effects in an early phase by environmental genotoxins [26,27]. In human populations, alkaline comet assays can be easily assessed in diverse cellular lines and exfoliated epithelial cells (e.g., oral, urothelial, nasal) to obtain a measure of genome damage induced in vivo [28,29]. Primary DNA damage is considered to be an important initial event in carcinogenesis and in the development of diseases [30].

The alkaline comet assay (single-cell gel electrophoresis) has become the preferred test for the qualitative and quantitative assessment of DNA damage in single cells, and is capable of detecting DNA single- and double-strand breaks, alkali-labile sites, incomplete excision repair sites, and genomic structural discontinuities [31]. Given the recognized effects of heavy metal exposure on children’s health, this research evaluated DNA damage in a group of 101 children (6 to 12 years old) living beside the El Fraile mine tailings, and a nonexposure group consisting of 101 nonexposed children of the same ages from Chilpancingo City, Guerrero, Mexico, who had never lived near mine zones or been exposed to heavy metals, through the two biomarkers of exposure, urinary 8-OHdG adduct and comet alkaline assay in exfoliated buccal mucosa cells.

## 2. Materials and Methods

### 2.1. Study Areas

El Fraile mining tailings are located between the parallels 18°30′ and 18°35′ and meridians 99°32′30″ and 99°40′ in the southeastern part of Taxco de Alarcón City. The El Fraile community is located beside the El Fraile mine tailings, 100 m from the abandoned El Fraile mine; 15 km from Taxco of Alarcon City in Northern Guerrero State, México (Figure 1).

### 2.2. Study Populations

The exposure group consisted of 101 elementary school children living in the El Fraile rural community among mining tailings, 100 m from the abandoned El Fraile mine. The control group also consisted of 101 elementary school children from Chilpancingo City in South Guerrero State, México, who had never lived near or in mining zones (Figure 1). The characteristics of both groups are described in Table 1. The Ethics Committee of the Faculty of Medicine from Universidad Nacional Autonoma de Mexico approved the research procedures used in this study, and a clinical questionnaire was administered to parents aiming to obtain information on food consumption habits, leisure activities, and current and former occupations of the parents. All donors were otherwise healthy. The parents were provided with information about the genetic research and informed that access to exfoliated buccal mucosa cells and urine samples are noninvasive methods for biological specimen collection and are relatively safe. Written informed consent was obtained from the parents of each participant in accordance with the Declaration of Helsinki Ethical Principles (2008). The age range of the exposure and control groups was 6 to 12 years old. The exposure group consisted of 56 girls and 45 boys, and the control group included 52 girls and 49 boys (Table 1). Before biological sample collection, exclusion criteria were applied for both exposure and control groups, and parents were asked for detailed information regarding age, diet, habits, present or previous history of any disease or exposure to diagnostic X-rays, medical examinations, and years of living in their current home. None of the healthy children used any medications during the study period.

### 2.3. Oral Mucosa Samples Collection

Exfoliated buccal mucosa samples were collected from the exposure and control children groups with a sterile spoon for each child and kept in sterile microtubes with phosphate-buffered saline (PBS; pH 7.4; GIBCO^TM^ Thermo Fisher Scientific, Grand Island, New York, NY, USA). All samples were kept on ice and immediately transferred to the Geosciences Laboratory (Escuela Superior de Ciencias de la Tierra, Universidad Autónoma de Guerrero), and centrifuged at 1500 rpm for 10 min. The cellular layer was diluted with PBS and immediately assessed in the alkaline comet assay [29,31].

### 2.4. Urine Collection

Urine samples of all children (exposure and control groups) were collected from the first morning void in polypropylene containers (25–50 mL), kept on ice, transferred to the Environmental Toxicology laboratory, and centrifuged at 800 g for 10 min. The supernatant was stored at −80 °C until analysis of the urinary 8-OHdG levels by enzyme-linked immunosorbent assay [26].

### 2.5. Alkaline Comet Assay

**Figure 2:**
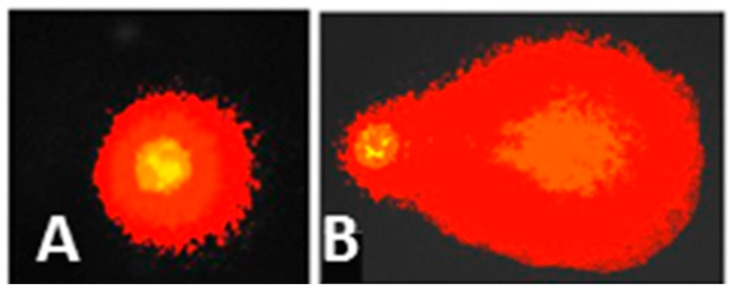
Nuclei without comet (without DNA damage) (**A**) and with comet (with DNA damage) (**B**) from exfoliated buccal mucosa cells.

Exfoliated buccal mucosa cells were kept in a sterile microtube with 180 µL low melting point agarose (0.5%, 37 °C) (Sigma-Aldrich, Darmstadt, Germany), mixed gently, and 90 µL were placed on a slide coated with a thin layer of normal melting point agarose (1 %, 37 °C) (Sigma-Aldrich, Darmstadt, Germany) and covered with a coverslip. Two slides were utilized for each child’s sample. The slides were maintained at 4 °C for 3–5 min to solidify the agarose. The coverslip was then carefully removed, and the slides were immersed in a staining jar containing a freshly prepared cold lysis solution (2.5 M NaCl, 100 mM EDTA, 10 mM Tris, 1% Triton X − 100 and 10% DMSO, pH 10) (Amresco, Solon, OH, USA) at 4 °C for 1 h. The slides were placed in a horizontal electrophoresis chamber (Thermo Fisher Scientific, USA) containing freshly prepared cold electrophoresis alkaline buffer (300 mM NaOH and 1 mM EDTA, pH 13) (Amresco, Solon, OH, USA) for 20 min to unwind the DNA. Electrophoresis was performed at 25 V (0.7 V/cm) and 300 mA for 20 min in darkness to prevent additional DNA damage. The slides were washed three times with freshly prepared neutralization buffer (0.4 M Tris, pH 7.5) for 5 min, fixed with cold absolute methanol for 5 min, and air-dried at room temperature. Then, 60 µL GelRed (4 µg/mL; Biotium, Inc., Fremont, CA, USA) was added to each slide to stain the DNA. The slides were labeled with a code that was unfamiliar to the viewer and examined using a Nikon Eclipse fluorescence microscope equipped with an excitation filter (515–560 nm) and a barrier filter (590 nm). To visualize the DNA damage, the slides were observed at 40× magnification using Comet IV Software (Perceptive Instruments, Bury St Edmunds, UK). Three parameters were used to determine genotoxicity: (a) tail length (the distance between the first and last DNA fragments); (b) tail moment (the tail length weighted by the percentage of tail DNA); and tail intensity (the percentage of DNA in the tail) in 50 randomly selected nuclei on each slide (two slides per child for a total of 100 nuclei) (Figure 2). Comets with completely fragmented DNA (hedgehog-like figures with no apparent head) were excluded from the evaluation [32].

### 2.6. Detection of Urinary 8-OHdG Levels by Enzyme-Linked Immunosorbent Assay (ELISA)

The 8-OHdG adduct levels were measured in a 100 µL urine sample from each child in triplicate using an 8-OHdG EIA kit (Cayman Chemicals, Ellsworth Rd, Ann Arbor, MI, USA) for ELISA, following the procedure suggested by the manufacturers. First, 50 µL EIA buffer, 50 µL 8-hydroxy-2′-deoxy-guanosine tracer AChE, and 50 µL 8-OHdG monoclonal antibody were added to each well, except the blank. The plate was covered with plastic film and incubated for 2 h at 4 °C in an orbital shaker (Rocker 25 Labnet). Then, the wells were washed five times with wash buffer, and 200 µL Ellman’s Reagent was added. The plate was covered with plastic film and incubated for 2 h at room temperature in an orbital shaker (Rocker 25 Labnet) in darkness. The plates were read at a wavelength of 450 nm in an ELISA Reader (Elx800 BioTec) using Gen 5 ELISA software. The data obtained were processed in a calculus sheet (provided by Cayman Chemicals), and the 8-OHdG concentrations were obtained with an 8-OHdG standard curve (10.3–3000 ng/mL) and expressed in ng/mL [26].

### 2.7. Statistical Analysis

The results of the tail length (µm), tail moment (%), tail intensity (%), and urinary 8-OHdG levels were reported as the mean ± SEM of 101 children, for range of age and gender of the exposure and control groups. The data were analyzed using Student’s *t*-test analysis to determine statistical significance between the exposure and control groups at *p* < 0.05. The relationship between the three genotoxic parameters and the level of 8-OHdG with age and gender were determined using a MANOVA test, *p* < 0.01 (Spearman’s rank correlation analysis), with SPSS Statistics.

## 3. Results

### 3.1. Characteristics of the Participant Children

The sociodemographic and dietary data from the exposure and control groups are shown in Table 1. The data suggest that food, air, soil, and water are major sources of heavy metal exposure in children who live near the El Fraile tailings that contribute to increased DNA damage. School outdoor areas in which schoolchildren play also constitute important outdoor sources of heavy metals at schools. Parents also reported using water from mine tailings to wash clothing, and the Cacalotenango River in the dry season was sometimes used as drinking water or to wash clothing (Figure 1). Additionally, in the indoor air of classrooms and in the respective outdoor areas, the reported heavy metal levels were strongly influenced by their proximity to the mine tailings. Outdoor air penetration impacts the indoor air of classrooms, mainly if natural ventilation is used throughout the day (including during class hours). Ambient air infiltration is strongly dependent on the ventilation rates of the classrooms in the dry season.

### 3.2. DNA Damage in Buccal Mucosa Cells in the (El Fraile)-Exposed and (Chilpancingo)-Control Groups

The results of the genotoxic analysis of the buccal mucosa cells from the exposure and control groups are shown in Table 2. There was a significant increase in the mean of the three genotoxic parameters of tail intensity (TI), tail moment (TM), and tail length (TL) in the exposed child group compared with the control group (Student’s *t*-test, *p* < 0.05). Comparing genotoxic parameters between exposure and control groups by age and gender, we observed that there were significant differences in the three genotoxic parameters (Table 2 and Table 3). However, when we compared three genotoxic parameter means from the exposure group by age and gender, we found that there were no significant differences in the three genotoxic parameters in the children in the 6–7 years group when compared with the children in the 10–12 years group (MANOVA, *p* < 0.01) (Table 2). However, the children aged 8–9 years had a greater increase in the mean of the three genotoxic parameters than the children aged 6–7 and 10–12 years (MANOVA, *p* < 0.01). Comparing the genotoxic effect with gender, we found that the boys’ exposure group had a higher increase in all three genotoxic parameters than the girls (*p* < 0.05) (Table 3). There was a positive correlation between sex and age, and a significant increase in DNA damage by Spearman´s rank correlation analysis (r = 0.65) (Table 3).

### 3.3. Oxidative DNA Damage in the Exposure and Control Groups

The mean urinary 8-OHdG levels from the exposure and control groups are presented in Table 4. There was a significant increase in the mean urinary 8-OHdG level (5.65 ± 0.33 ng/mL) in the exposure group compared to the control group value (1.64 ± 0.88 ng/mL) (Student’s *t*-test, *p* < 0.05). There was a statistically significant increase in the mean urinary 8-OHdG levels in children aged 8–9 years (6.95 ± 0.24 ng/mL) compared with the urinary 8-OHdG adduct means from children aged 6–7 (5.15 ± 0.2 ng/mL) and 10–12 years (4.35 ± 0.54 ng/mL) from the exposure group (MANOVA, *p* < 0.01) (Table 4). Comparing the 8-OHdG adduct mean level of the girls with the boys from the exposure group, we observed that oxidative DNA damage was significantly greater in the boys (6.88 ± 0.12 ng/mL) than in the girls (5.18 ± 0.24 ng/mL) (Table 5). There was a positive correlation between sex and age, and a significant increase in oxidative DNA damage with Spearman´s rank correlation analysis (r = 0.72) (Table 5).

## 4. Discussion

Previous studies have shown that the metal concentrations in the El Fraile mine tailings are very high because of geochemical, physicochemical, and environmental factors [2,5]. Heavy metals such as As (140–3627 mg/kg), Cd (0.5–434 mg/Kg), Pb (148–0900 mg/kg), Cu (0.002–55%), Zn (0.021–3.86%), and Fe (2.4–35.7%) have been identified in this zone [1,2]. El Fraile leachates, which are used as domestic water, typically exceed the Mexican Drinking Water Guidelines for sulfate, hardness, Fe, Mn, Pb, and As, while acidic leachates exceed the Mexican Guidelines for Industrial Discharge Waters for pH, As (<0.01–12.0 mg/L), Fe (0.025–2352 mg/L), Mn (0.1–732 mg/L), Zn (<0.025–1465 mg/L)1), and Pb (<0.01–0.351 mg/L) [1,2]. Moreover, a metal analysis of maize plants reported that Pb was in the range of 25.2 to 300.9 mg/kg, and Zn was in the range of 88.9 to 504.8 mg/ kg; the aerial part concentrations were in the range of 15.5 to 555.6 mg/kg for Zn and 2.2 to 10.8 mg/kg for Pb, which represent other risk factors since corn is the food base of the Mexican diet. High heavy metal levels in vegetal tissues, air, soil, and drinking water in the El Fraile area clearly indicate that the general population is highly exposed to heavy metals [33].

Developing children are the most sensitive population with regard to adverse effects from these environmental genotoxins [7,10]. In this regard, this study is the first to evaluate DNA damage using the alkaline comet assay in exfoliated buccal mucosa cells from 101 children (56 girls and 45 boys) aged 6–7, 8–9, and 10–12 years living beside the El Fraile mine tailings. In addition, oxidative DNA damage was quantified with the detection of urinary 8-OHdG adduct levels by ELISA [20,34]. We also evaluated a control group consisting of 101 children (52 girls and 49 boys) from Chilpancingo City, Guerrero, Mexico, within the same age ranges, who had never lived near or in mining zones. The results of the genotoxic analysis evidenced a higher increase in DNA damage in exfoliated buccal mucosa cells of the exposure children group compared with the control group (*p* < 0.05), demonstrated by a high increase in the three genotoxic parameter means (TI, TM, and TL) (*p* < 0.05). We applied a MANOVA to the data on the three genotoxic parameters in children aged 6–7, 8–9, and 10–12 years from the exposure group with respect to sex and age. We found that boys aged 8–9 years had higher DNA damage than girls (*p* < 0.01). Moreover, we found a positive Spearman’s rank correlation between increased DNA damage and sex (boys) and age (8–10 years) in the exposure group (r = 0.65; *p* < 0.01). 

Our results agree with other genotoxic studies in children living near mining zones. Children exposed to Pb and As from the Villa de la Paz mine, Mexico, had significant increases in the tail length (67.6 μm) and the tail moment (6.08%) in human peripheral blood lymphocytes [35]. Another study also reported a significant increase in DNA damage (49.1–61.8%), with tail lengths ranging from 25.3 to 29.2 μm in peripheral blood lymphocytes from children aged 6 to 11 years exposed to soil and water contaminated with Pb and AS in the region of Lagunera, Mexico [36]. Children aged 4 to 11 years living 200 km away from a mining zone and exposed to Pb and As in Luis Potosi, Mexico, evidenced a significant increase in the tail moment (5.2 μm) and tail length (3.5 μm) in peripheral lymphocytes [37]. In children aged 6 to 11 years who drank water contaminated with Pb and As, a significant increase was observed in the tail moment (>4.8) of lymphocytes [38]. A reduction in telomere length and mitochondrial DNA were observed in children (6 to 15 years old) exposed to As and Pb that lived in an industrial area in Salamanca, Mexico [39].

This study evidenced that children of all ages from the exposure group showed a significant increment in DNA damage in relation to the control group, but the children aged 8–9 years had a higher increment in their genotoxic parameters (*p* < 0.05) than children aged 6–7 and 10–12 years from the same exposure group (*p* < 0.01). In peripheral blood cells from 9-year-old children exposed to Pb in Poland, a significant increase was found in the frequency of micronuclei [40], as well as an increase in DNA strand breaks in children living around a waste incinerator [41]. These results possibly indicate that children, especially boys aged 8 to 9 years old, are more vulnerable to heavy metal exposure than children aged 6–7 and 10–12 years, due to the childrens’ physiological conditions and exposure time; they consume more food and have high gastrointestinal uptake and other physiological factors (detoxification system immaturity) [42,43]. Furthermore, boys in these age ranges spend more time outside, and they have more environmental exposure to heavy metals by air inhalation, soil dermal contact or absorption, or by ingestion of contaminated water and food than girls [10,44]. Previous studies have demonstrated that children’s unique behaviors and metabolic rate often place them at risk for absorption of higher doses of heavy metals in contaminated environments in comparison to adults [9,45]. Many studies have also indicated that direct soil exposure and hand-to-mouth activity are the principal routes that contribute to heavy metal exposure in children [43,46]. Unintentional ingestion of heavy metals may lead to a considerably higher dose than an adult because of the greater intake of food or fluids per pound of body weight [42]. Heavy metals are introduced into the atmosphere by air particles, into the food chain by deposition on crop plants, and into drinking water, as well as by soil dust inhalation [1,2,3,4,5,7,33]. Regarding heavy metals in body tissues, the brain, kidneys, liver, heart, bones, and blood are the main target organs for heavy metal accumulation in children [10,11,15]. Most likely, children living in the El Fraile rural community are exposed to a high heavy metal mix that produces ROS or NOS that directly or indirectly interact with their DNA, which has caused its fragmentation, as evidenced by greater comets in buccal mucosa cell nuclei.

In parallel, we analyzed oxidative DNA damage through urinary 8-OHdG adduct levels in the children from the exposure and control groups, and our results showed that the urinary 8-OHdG level was three times higher in the exposure group than in the control group (*p* < 0.01). When we applied a MANOVA test to urinary 8-OHdG mean levels in the children from the 6–7, 8–9, and 10–12 age ranges and by sex from the exposure group, we observed that the boys aged 8–10 years had five times more 8-OHdG adduct than boys from the nonexposure group (MANOVA, *p* < 0.01). Furthermore, we found a positive Spearman’s rank correlation between increased oxidative DNA damage and sex (boys) and age (8–9 years) in the exposure group (r = 0.72; *p* < 0.01). Our results are consistent with various published studies, in which children exposed to chromium and a heavy metal mix showed a significant increase in urinary 8-OHdG levels [47]. Chronic exposure to low levels of Cd produces oxidative stress in children [48]. Children within the age range of 3 to 6 years with high Pb exposure from an electronic waste recycling center showed a significant increase in urinary 8-OHdG levels [41]. In adolescents aged 12–14 years old, exposure to heavy metals in Milazzo-Valle del Mela (Sicily, Italy) led to higher levels of urinary 8OHdG adduct [49]. A significant increase in urinary 8-OHdG levels in children exposed to As [50] and salivary 8-OHdG levels in children 5–7 years old exposed to water contaminated with As [51] was reported. An increase was observed in the 8-OHdG adduct in children 5–8 years old with a high blood lead (Pb) level, and in children 6–8 years old with high Cd levels from Montevideo, Uruguay [52,53]. In children living near the petrochemical industry in Central Taiwan, elevated 8-OHdG levels were associated with copper, arsenic, strontium, cadmium, and mercury exposure [12].

In our study, urinary 8-OHdG levels detected by ELISA in both the exposure and control groups agreed with what has been reported in other studies [34,52]. Our results were supported by high levels of heavy metals and metalloids reported in blood and urine samples in children from the El Fraile community within the same age ranges that had been included in our study [3]. Our results clearly indicate that the bodies of the children had experienced an increase in oxidative DNA damage due to high environmental exposure to heavy metals, which produced a greater increase in ROS, such as OH•, which are capable of oxidizing mainly guanine to produce an 8-OHdG adduct, which is excreted in urine, as demonstrated by higher urinary 8-OHdG levels. 

8-Hydroxy-deoxyguanosine (8-OHdG) is one of the most widely used biomarkers for detecting oxidative DNA damage [22,23]. The formation of DNA adducts, one of the earliest molecular changes caused by environmental chemicals, may promote the initiation and progression of carcinogenesis [54,55,56]. Furthermore, these data supported the hypothesis that children who live in the El Fraile community have higher oxidative stress in their body, which consequently reduces their genome repair mechanisms, and a low capacity of endogenous antioxidants in the child’s body makes them more vulnerable to genome oxidation. Several studies have shown that populations that are chronically exposed to heavy metals, such as Cd, Ni, Pb, and Hg, have a significant increase in oxidative stress [11,12,16,56] and DNA damage, such as strand breaks, mutations, chromosomal aberrations, oxidation of their bases, and decreased endogenous antioxidant levels [15,50,53,54,55]. Moreover, an increase in ROS and NOS can modify cellular signaling pathways, transcription factors, and gene regulation, which are mechanisms that play a crucial role in carcinogenesis and immunosuppression in childhood [57,58]. Chronic exposure to drinking water containing high levels of metals and metalloids is associated with various gastrointestinal diseases [46], diabetes, and cardiovascular diseases [15,30].

It is worth mentioning that our study had a few limitations. First, we could not detect heavy metal concentrations in urine samples or in blood in either the exposure or control groups to associate them with DNA strand breaks and urinary 8-OHdG levels, or with sociodemographic variables. However, it is important to mention that this study is the first to evaluate DNA damage with two early genetic biomarker exposures to environmental pollution using noninvasive methods, such as buccal mucosa cells and urine samples, to demonstrate that children from the El Fraile rural community with high heavy metal pollution are at a high health risk; the results can support the prevention of developmental diseases.

## 5. Conclusions

The results of this study demonstrate that children (boys and girls) living in the El Fraile rural community near the El Fraile mine tailings are exposure to an area heavily polluted by toxic heavy metal mixes and are at a high health risk, since the genotoxic results demonstrated an increase in DNA damage (genome strand breaks and oxidation) in the exposure group relative to the control group through the two genetic biomarkers of alkaline comet assay and urinary 8-OHdG adduct. However, the boys were shown to have more DNA damage than the girls. Nonetheless, diverse factors can contribute to genotoxic actions due to the exposure of children to heavy metals, such as lifestyle, water, soil, and food contamination, and the synergistic or additive actions of heavy metal mixes.

## Figures and Tables

**Table 1:** Sociodemographic characteristics in the (El Fraile, Taxco de Alarcón)-exposure and (Chilpancingo)-control groups.

Variable	Control Group ^a^	Exposure Group ^a^
Age ranges	6–12	6–12
Gender	52 girls 49 boys	56 girls 45 boys
Living area	Urban	Rural
Diet	Not special	Not special
Lifestyle Children play in garden	80%	85%
Type of drinking water and zone of residence (close to or far from mine tailings)	Bottled 60%	Bottled 100%
Frequency of children’s contact with soil Every day	10%	85%
Frequency of consuming fruits and edible vegetables grown on site	No	100%
Frequency of Cacalotenango water river use	None	100%
Frequency of rain water use Every day 2–3 times/week Once a week	None	70%
Children consuming meat from animals grown on site	None	85%
Children consuming milk from cows grown on site	None	80%
Socioeconomic status (parental education level)	Elementary, high school	Elementary school

^a^ n = 101.

**Table 2:** Mean of the genotoxic parameters in buccal mucosa cells in the (El Fraile, Taxco de Alarcón)-exposure and (Chilpancingo)-control groups in Guerrero State, Mexico.

Children Groups (Age Ranges)	n	Tail Intensity (%)	Tail Moment (%)	Tail Length (µm)
		Mean ± SEM	Mean ± SEM	Mean ± SEM
Exposure	101	37.68 ± 4.59 *	19.79 ± 3.50 *	60.89 ± 6.18 *
6–7	29	45.41 ± 5.40 *	17.86 ± 5.07 *	56.32 ± 4.36 *
8–9	39	49.94 ± 4.51 **	24.54 ± 6.64 **	71.14 ± 4.98 **
10–12	33	39.94 ± 4.31 *	17.25 ± 4.95 *	55.81 ± 6.10 *
Control	101	18.38 ± 1.52	4.24 ± 1.54	36.59 ± 1.86
6–7	30	16.76 ± 1.44	3.91 ± 2.30	46.13 ± 4.84
8–9	39	18.81 ± 1.38	4.58 ± 2.77	34.27 ± 4.08
10–11	32	14.53 ± 1.66	4.16 ± 2.65	44.01 ± 2.03

* Significant differences between exposure and control groups were obtained by a Student’s *t*-test, *p* < 0.05) and a MANOVA test ** *p* < 0.01.

**Table 3:** Mean of the genotoxic parameters by gender in buccal mucosa cells in the (El Fraile, Taxco de Alarcón)-exposure and (Chilpancingo)-control groups in Guerrero State, Mexico.

Gender	Children’s Groups	n	Tail Intensity (%)	Tail Moment (%)	Tail Length (µm)
			Mean ± SEM	Mean ± SEM	Mean ± SEM
	Exposure				
Girls		56	44.33 ± 5.26 *	22.01 ± 5.49 *	58.73 ± 5.71 *
Boys		45	58.70 ± 6.44 **	28.93 ± 3.01 **	68.13 ± 4.99 **
	Control				
Girls		52	14.33 ± 5.04	5.01 ± 4.49	28.73 ± 4.71
boys		49	17.70 ± 2.88	6.29 ± 3.01	24.13 ± 6.11

* Significant differences among exposure and control groups were obtained by an analysis with Student´s *t*-test *p* < 0.05 and a MANOVA test ** *p* < 0.01.

**Table 4:** Mean of the urinary 8-OHdG levels in the (El Fraile, Taxco de Alarcón)-exposure and (Chilpancingo)-control groups in Guerrero State, Mexico.

Age Ranges	Children Groups	(n)	8-OHdG Level (ng/mL) Mean ± SEM
	Exposure	101	5.65 ± 0.33 *
6–7		29	5.15 ± 0.67 *
8–9		39	6.95 ± 0.24 **
10–12		33	4.35 ± 0.54 *
	Control	101	1.64 ± 0.88
6–7		30	1.13 ± 0.24
8–9		39	1.88 ± 0.12
10–12		32	1.74 ± 0.30

* Significant differences between exposure and control groups were obtained by an analysis with Student’s *t*-test, *p* < 0.05, and a MANOVA test, ** *p* < 0.01.

**Table 5:** Means of urinary 8OHdG (ng/mL) levels by gender in the (El Fraile, Taxco de Alarcón)-exposure and (Chilpancingo)-control groups, in Guerrero State, Mexico.

Gender	Children Groups	n	8-OHdG Level (ng/mL) Mean ± SEM
	Exposure		
Girls		56	5.18 ± 0.24 *
Boys		45	6.88 ± 0.12 **
	Control		
Girls		52	1.73 ± 0.24
Boys		49	1.38 ± 0.08

* Significant differences between exposure and control groups were obtained by an analysis with Student´s *t* test, *p* < 0.05, and a MANOVA test ** *p* < 0.01.

## Data Availability

Not applicable.

## References

[1] Talavera-Mendoza O., Armienta-Hernández M.A., García-Abundis J., Flores-Mundo N. (2006). Geochemistry of leachates from the El Fraile sulfide tailings piles in Taxco, Guerrero, southern Mexico. Environ. Geochem. Health.

[2] Romero F.M., Nuñéz M.E., Gutiérrez M.A., Armienta A.E., Cineros-Gómez A.E. (2011). Evaluation of the potential of indigenous calcareous shale for neutralization and removal of arsenic and heavy metals from acid mine drainage in the Taxco mining area, Mexico. Arch. Environ. Contam. Toxicol..

[3] Moreno M.E., Acosta-Saavedra L.C., Meza-Figueroa D., Vera E., Cebrian M.E., Ostrosky-Wegman P., Calderón-Aranda E.S. (2010). Biomonitoring of metal in children living in a mine tailings zone in Southern Mexico: A pilot study. Int. J. Hyg. Environ. Health.

[4] Soto-Ríos M.L., Rothenberg S., Gonsebatt M.E., Talavera-Mendoza O. (2010). Cytogenotoxicity in uroepithelial cells of women exposed to mercury in a mining area. Occup. Environ. Med..

[5] Armienta M.A., Talavera O., Morton O., Barrera M. (2003). Geochemistry of Metals from Mine Tailings in Taxco, Mexico. Bull. Environ. Contam. Toxicol..

[6] de Burbure C., Buchet J.P., Bernard A., Leroyer A., Nisse C., Haguenoer J.M., Bergamaschi E., Mutti A. (2003). Biomarkers of renal effects in children and adults with low environmental exposure to heavy metals. J. Toxicol. Environ. Health A.

[7] Al Osman M., Yang F., Massey Y.I. (2019). Exposure routes and health effects of heavy metals on children. Biometals.

[8] Bellinger D.C. (2008). Very low lead exposures and children’s neurodevelopment. Curr. Opin. Pediatr..

[9] Dunn A.M., Burns C., Satiler B. (2003). Environmental Health children. J. Pediatr. Health Care.

[10] Wright R.O. (2017). Environment, susceptibility windows, development, and child health. Curr. Opin. Pediatr..

[11] Granot E., Kohen R. (2004). Oxidative stress in childhood in health and disease states. Clin. Nutr..

[12] Killian B., Yuan T.H., Tsai C.H., Chiu T.H.T., Chen Y.H., Chan C.C. (2020). Emission-Related Heavy Metal Associated with Oxidative Stress in Children: Effect of Antioxidant Intake. Int. J. Environ. Res. Public Health.

[13] Romaniuk A., Lyndin M., Sikora V., Lyndina Y., Romaniuk S., Sikora K. (2017). Heavy metals effect on breast cancer progression. J. Occup. Med. Toxicol..

[14] Loft S., Svoboda P., Kasai H., Tjønneland A., Vogel U., Møller P., Overvad K., Raaschou-Nielsen O. (2006). Prospective study of 8-oxo-7,8-dihydro-2′-deoxyguanosine excretion and the risk of lung cancer. Carcinogenesis.

[15] Korashy H.M., Attafi I.M., Famulski K.S., Bakheet S.A., Hafez M.M., Alsaad A.M.S., Al-Ghadeer A.R.M. (2017). Gene expression profiling to identify the toxicities and potentially relevant human disease outcomes associated with environmental heavy metal exposure. Environ. Pollut..

[16] Ercal N., Gurer-Orhan H., Aykin-Burns N. (2001). Toxic metals and oxidative stress part I: Mechanisms involved in metal induced oxidative damage. Curr. Top. Med. Chem..

[17] Marnett L.J. (2000). Oxyradicals and DNA damage. Carcinogenesis.

[18] Cooke M.S., Olinski R., Loft S. (2008). Measurement and meaning of oxidatively modified DNA lesion in Urine. Cancer Epidemiol. Biomark. Prev..

[19] Hartwig A., Schwerdtle T. (2002). Interactions by carcinogenic metal compounds with DNA repair processes: Toxicological implications. Toxicol. Lett..

[20] Drury J.A., Jeffers G., Cooke R.W. (1998). Urinary 8-hydroxydeoxyguanosine in infants and children. Free Radic. Res..

[21] Poulsen H.E., Nadal L.L., Broedbaek K., Nielsen P.E., Weimann A. (2014). Detection and interpretation of 8-oxodG and 8-oxoGua in urine, plasma and cerebrospinal fluid. Biochim. Biophys. Acta.

[22] Cheng K.C., Cahill D.S., Kasai H., Nishimura S., Loeb L.A. (1992). 8-Hydroxyguanine, an abundant form of oxidative DNA damage causes G-t and A-C substitutions. J. Biol. Chem..

[23] Chiou C.C., Chang P.Y., Chan E.C., Wu T.L., Tsao K.C., Wu J.T. (2003). Urinary 8-hydroxydeoxyguanosine and its analogues as DNA marker of oxidative stress: Development of an ELISA and measurement in both bladder and prostate cancers. Clin. Chim. Acta.

[24] Bernard A. (2008). Biomarkers of metal toxicity in population studies: Research potential and interpretation issues. J. Toxicol. Environ. Health. Part A.

[25] Calderón-Segura M.E., Gómez-Arroyo S., Villalobos-Pietrini R., Espinosa-Ramírez M. (1999). In vivo and in vitro promutagen activation by *Vicia faba* of thiocarbamate herbicides molinate and butylate to products inducing sister chromatid exchanges in human lymphocyte cultures. Mutat. Res..

[26] Rodríguez-Romero M.I., Gómez-Arroyo S., Villalobos-Pietrini R., Martínez-Valenzuela C., Calderón-Ezquerro M.C., Cortés-Eslava J., Arenas-Huertero F., Calderón-Segura M.E. (2012). Evaluation of 8-hidroxy-2′-deoxiguanosine (8-OHdG) adduct levels and DNA strand breaks in human peripheral lymphocytes in vitro exposed to polycyclic aromatic hydrocarbons with or without animal metabolic activation. Toxicol. Mech. Methods.

[27] Tsukahara H. (2007). Biomarkers for oxidative stress: Clinical application in pediatric medicine. Curr. Med. Chem..

[28] Martínez-Valenzuela C., Waliszewski S.M., Amador-Muñoz O., Meza E., Calderón-Segura M.E., Zenteno E., Huichapan-Martínez J., Caba M., Félix-Gastélum R., Longoria-Espinoza R. (2016). Aerial pesticide application causes DNA damage in pilots from Sinaloa, Mexico. Environ. Sci. Pollut. Res..

[29] Carbajal-López Y., Gómez-Arroyo S., Villalobos-Pietrini R., Calderón-Segura M.E., Martínez-Arroyo A. (2016). Biomonitoring of agricultural workers exposed to pesticide mixtures in Guerrero state, Mexico, with comet assay and micronucleus test. Environ. Sci. Pollut. Res..

[30] Zhushan F., Shuhua X. (2020). The effects of heavy metals on human metabolism. Toxicol. Mech. Methods.

[31] Singh N.P., McCoy M.T., Tice R.R., Schneider E.L. (1988). A simple technique for quantification of low levels of DNA damage in individual cells. Exp. Cell Res..

[32] Calderón-Segura M.E., Gómez-Arroyo S., Cortés-Eslava J., Martínez-Valenzuela C., Mojica-Vázquez L.H., Sosa-López M., Flores-Ramírez D., Romero-Velázquez Z.E. (2018). In Vitro cytotoxicity and genotoxicity of Furia ®180 SC (Zeta-cypermethrin) and Bulldock 125®SC (β-Cyfluthrin) pyrethroid insecticides in human peripheral blood lymphocytes. Toxicol. Mech. Methods.

[33] Díaz-Villaseñor E. (2006). Transferencia de Metales Entre Suelo y Plantas de Maíz (*Zea mays* L.), Sembradas en Terrenos Impactados Por Jales Mineros en la Región de Taxco, Guerrero. Master’s Thesis.

[34] Breton J., Sichel F., Bianchini F., Prevost V. (2003). Measurement of 8-Hydroxy-2′deoxyguanosine by a commercially available ELISA test: Comparison with HPLC/electrochemical detection in calf thymus DNA and determination in human serum. J. Anal. Lett..

[35] Yáñez L., García-Nieto E., Rojas E., Carrizales L., Mejía J., Calderón J., Razo I., Díaz-Barriga F. (2003). DNA damage in blood cells from children exposed to arsenic and lead in a mining area. Environ. Res..

[36] Méndez-Gómez J., García-Vargas G.G., López-Carrillo L., Calderón-Aranda E.S., Gómez A., Vera E., Valverde M., Cebrián M.E., Rojas E. (2008). Genotoxic Effects of Environmental Exposure to Arsenic and Lead on Children in Region Lagunera, Mexico. Ann. N. Y. Acad. Sci..

[37] Jasso-Pineda Y., Díaz-Barriga F., Calderón J., Yáñez L., Carrizales L., Pérez-Maldonado I.N. (2012). DNA damage and decreased DNA repair in peripheral blood mononuclear cells in individuals exposed to arsenic and lead in a mining site. Biol. Trace Elem. Res..

[38] Jasso-Pineda Y., Díaz-Barriga F., Yáñez-Estrada L., Pérez-Vázquez F.J., Pérez-Maldonado I.N. (2015). DNA damage in Mexican children living in high-risk contaminated scenarios. Sci. Total Environ..

[39] Alegría-Torres J.A., Pérez-Rodríguez R.Y., García-Torres L., Costilla-Salazar R., Rocha-Amador D. (2020). Exposure to arsenic and lead in children from Salamanca México, effects on telomeric lengthening and mitochondrial DNA. Environ. Sci. Pollut. Res. Int..

[40] Kapka L., Baumgartner A., Siwińska E., Knudsen L.E., Anderson D., Mielzyńska D. (2007). Environmental lead exposure increases micronuclei in children. Mutagenesis.

[41] Xu X., Liao W., Lin Y., Dai Y., Shi Z., Huo X. (2018). Blood concentrations of lead, cadmium, mercury and their association with biomarkers of DNA oxidative damage in preschool children living in an e-waste recycling area. Environ. Geochem. Health.

[42] Moya J., Bearer C.F., Etzel R.A. (2004). Children’s behavior and physiology and how it affects exposure to environmental contaminants. Pediatrics.

[43] Sly J.L., Carpenter D.O. (2012). Special vulnerability of children to environmental exposures. Rev. Environ. Health.

[44] Neri M., Bonassi S., Knudsen L.E., Sram R.J., Holland N., Ugolini D., Merlo D.F. (2006). Children’s exposure to environmental pollutants and biomarkers of genetic damage I. Overview and critical issues. Mutat. Res..

[45] Engström K.S., Vahter M., Lindh C., Teichert F., Singh R., Concha G., Nermell B., Farmer P.B., Strömberg U., Broberg K. (2010). Low 8-oxo-7,8-dihydro-2-oxi-deoxyguanosine levels and influence of genetic background in an Andean population exposed to high levels of arsenic. Mutat. Res..

[46] Kim J.J. (2004). Ambient air pollution: Health hazards to children. Pediatrics.

[47] Sughis M., Nawrot T.S., Haufroid V., Nemery B. (2012). Adverse Health Effects of Child Labor: High Exposure to Chromium and Oxidative DNA Damage in Children Manufacturing Surgical Instruments. Environ. Health Perspect..

[48] Kippler M., Hossain M.B., Lindh C., Moore S.E., Kabir I., Vahter M., Broberg K. (2012). Early life low-level cadmium exposure is positively associated with increased oxidative stress. Environ. Res..

[49] Pizzino G., Bitto A., Interdonato M., Galfo F., Irrera N., Mecchio A., Pallio G., Ramistella V., De Luca F., Minutoli L. (2014). Oxidative stress and DNA repair and detoxification gene expression in adolescents exposed to heavy metals living in the Milazzo-Valledel Mela area (Sicily, Italy). Redox Biol..

[50] Xu Y., Wang Y., Zheng Q., Li X., Li B., Jin Y., Sun X., Sun G. (2008). Association of oxidative stress with arsenic methylation in chronic arsenic-exposed children and adults. Toxicol. Appl. Pharmacol..

[51] Hinhumpatch P., Navasumrit P., Chaisatra K., Promvijit J., Mahidol C., Ruchirawat M. (2013). Oxidative DNA damage and repair in children exposed to low levels of arsenic in utero and during early childhood: Application of salivary and urinary biomarkers. Toxicol. Appl. Pharmacol..

[52] Roy A., Queirolo E., Peregalli F., Mañay N., Martínez G., Kordas K. (2015). Association of blood lead levels with urinary F2-8α Isoprostane and 8-hydroxy-2-deoxy-Guanosine concentrations in first-grade Uruguayan children. Environ. Res..

[53] Kordas K., Roy A., Vahter M., Ravenscroft J., Mañay N., Peregalli F., Martínez G., Queirolo E.I. (2018). Multiple-metal exposure, diet, and oxidative stress in Uruguayan school children. Environ. Res..

[54] Fukuda M., Yamauchi H., Yamamoto H., Aminaka M., Murakami H., Kamiyama N., Miyamoto Y., Koitabashi Y. (2008). The evaluation of oxidative DNA damage in children with brain damage using 8-hydroxydeoxyguanosine levels. Brain Dev..

[55] Wong R.H., Kuo C.H., Hsu M.L., Wang T.Y., Chang P.I., Wu T.H., Huang S. (2005). Increased Levels of 8-Hydroxy-2′-Deoxyguanosine Attributable to Carcinogenic Metal Exposure among School children. Environ. Health Perspect..

[56] Pineda-Zavaleta A.P., García-Vargas G., Borja-Aburto V.H., Acosta-Saavedra L.C., Vera-Aguilar E., Gómez-Muñoz A., Cebrián M.E., Calderón-Aranda E.S. (2004). Nitric oxide and superoxide anion production in monocytes from children exposed to arsenic and lead in region Lagunera, Mexico. Toxicol. Appl. Pharmacol..

[57] Leonard S.S., Harris G.K., Shi X. (2004). Metal-induced oxidative stress and signal transduction. Free Radic. Biol. Med..

[58] Endo K., Miyashita Y., Sasaki H., Ebisuno M., Ohira M., Saiki A., Koide N., Oyama T., Takeyoshi M., Shirai K. (2006). Probucol and atorvastatin decrease urinary 8-hydroxy-2′-deoxyguanosine in patients with diabetes and hypercholesterolemia. J. Atheroscler. Thromb..

